# Feline Morbillivirus, a New Paramyxovirus Possibly Associated with Feline Kidney Disease

**DOI:** 10.3390/v12050501

**Published:** 2020-05-01

**Authors:** Eun Jin Choi, Victoria Ortega, Hector C. Aguilar

**Affiliations:** 1 Department of Microbiology, Cornell University, Ithaca, NY 14853, USA; ec759@cornell.edu; 2 Department of Microbiology and Immunology, College of Veterinary Medicine, Cornell University, Ithaca, NY 14850, USA; vo56@cornell.edu

**Keywords:** chronic kidney disease, feline morbillivirus, paramyxovirus, persistent infection, tubulointerstitial nephritis

## Abstract

Feline morbillivirus (FeMV) was first isolated in stray cats in Hong Kong in 2012. Since its discovery, the virus has been reported in domestic cats worldwide, including in Hong Kong, Japan, Italy, US, Brazil, Turkey, UK, Germany, and Malaysia. FeMV is classified in the *Morbillivirus* genus within the *Paramyxoviridae* family. FeMV research has focused primarily on determining the host range, symptoms, and characteristics of persistent infections in vitro. Importantly, there is a potential association between FeMV infection and feline kidney diseases, such as tubulointerstitial nephritis (TIN) and chronic kidney diseases (CKD), which are known to significantly affect feline health and survival. However, the tropism and viral entry mechanism(s) of FeMV remain unknown. In this review, we summarize the FeMV studies up to date, including the discoveries of various FeMV strains, basic virology, pathogenicity, and disease signs.

## 1. Introduction

### FeMV Belongs to Family Paramyxoviridae

Paramyxoviruses are enveloped, non-segmented, negative-sense, single-stranded RNA viruses [1,2]. They infect a large variety of mammalian hosts, such as humans, mice, pandas, hyenas, whales, bats, rats, dogs, and cats, as well as non-mammalian hosts, such as birds and reptiles [1,3,4,5,6,7]. Many pathogenic viruses within the *Paramyxoviridae* family significantly affect animal and human health. Examples include measles virus (MeV), mumps virus (MuV), Newcastle disease virus (NDV), Rinderpest virus (RPV), and the highly pathogenic zoonotic Hendra virus (HeV) and Nipah virus (NiV) [1,4,8,9]. Therefore, the outbreak of these viruses can cause critical human and veterinary health burden, as well economic damage to several livestock industries [10,11,12,13]. The *Morbillivirus* genus within the *Paramyxoviridae* family contains highly infectious animal viruses, including peste-des-petits-ruminants virus (PPRV), canine distemper virus (CDV), and cetacean morbillivirus (CeMV), which can cause severe and sometimes fatal systemic disorders [14,15,16]. In 2012, a previously unknown virus now named feline morbillivirus (FeMV, formerly abbreviated FmoPV), was discovered in Hong Kong to infect cats and subsequently classified in the *Morbillivirus* genus [1,17,18].

There are six genes in the paramyxovirus genome arranged in order 3′-N-P/V/W/C-M-F-HN/H/G-L-5′ [1]. The negative-strand RNA is tightly bound to the nucleocapsid (N) protein and forms a ribonucleoprotein complex (RNP) along with the large (L) RNA-dependent RNA polymerase and the phosphoprotein (P) [19]. The matrix protein (M) is a non-glycosylated peripheral membrane protein involved in virus particle assembly and budding [20,21]. Viral attachment and entry into target cells depend on two surface glycoproteins, the fusion (F) and the attachment or receptor binding [hemagglutinin–neuraminidase (HN) / hemagglutinin (H) / glycoprotein (G)] glycoproteins [3,22]. Paramyxovirus attachment glycoproteins bind to cellular receptors, such as neuraminidase–proteinaceous receptors (for HN), ephrinB2 and ephrinB3 (for G), and SLAM (also known as CD150, for H) [23,24,25,26]. After receptor binding, the two surface glycoproteins undergo conformational changes and trigger F to undergo the viral-cell membrane fusion cascade that results in viral entry. This process facilitates fusion of viral and host cell membranes and viral entry into host cells [27,28].

## 2. Discovery of Various FeMV Strains

**Figure 1:**
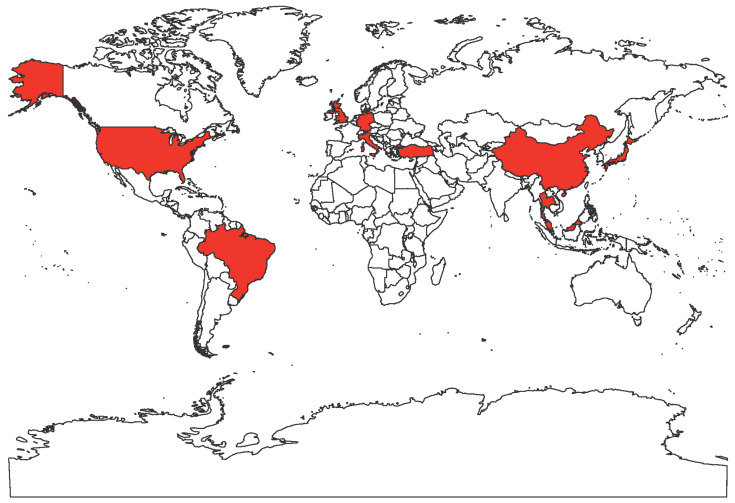
World map showing the countries with reported FeMV infections in felines in red.

FeMV is an emerging morbillivirus that has been isolated and studied by numerous research groups worldwide. Cats infected with FeMV have been detected in Hong Kong, Japan, Italy, United States, Brazil, Turkey, United Kingdom, Germany, Malaysia (Figure 1). FeMV RNA was first detected in 56 out of 457 stray cats (12.3%, 53 urine samples, four rectal swabs and one blood sample) by reverse transcription polymerase chain reaction (RT-PCR) utilizing consensus primers designed using the partial sequence of the morbillivirus L gene, a highly conserved sequence within the genome [1,10]. The three complete genome sequences (761U, 776U, and M252A) had less than 80% nucleotide identities to known paramyxoviruses [1]. The three genomes followed the characteristic paramyxovirus genome layout, the rule of six, and the herringbone nucleoprotein morphology [1,29,30]. Based on these observations and the phylogenetic analysis, the three strains were added to the *Morbillivirus* genus [1].

Since then, new FeMV strains have been continuously isolated from cat urine samples and identified by RT-PCR based on the partial L gene sequences. In 2014, viral RNA was detected in five out of 82 urine samples (6.1%) and one among ten blood samples (10%) in Japan. The six unknown viruses were determined to be FeMV strains (SE4, CL5, SE7, SE14, MS25, and MS26), as they shared 92–94% identity with the three viruses identified in Hong Kong [31]. Furthermore, three strains (SS1, SS2 and SS3) were isolated from 13 cat urine samples and had a 90–99% nucleotide similarity to the isolates from Hong Kong. SS3 showed an around 99% similarity to strain M252A [32]. Based on the high similarity between FeMV strains identified in Japan and Hong Kong, the researchers suggested a possible transmission of FeMV by unidentified vectors. For instance, infected cats may have been transported between the two countries [32].

**Figure 2:**
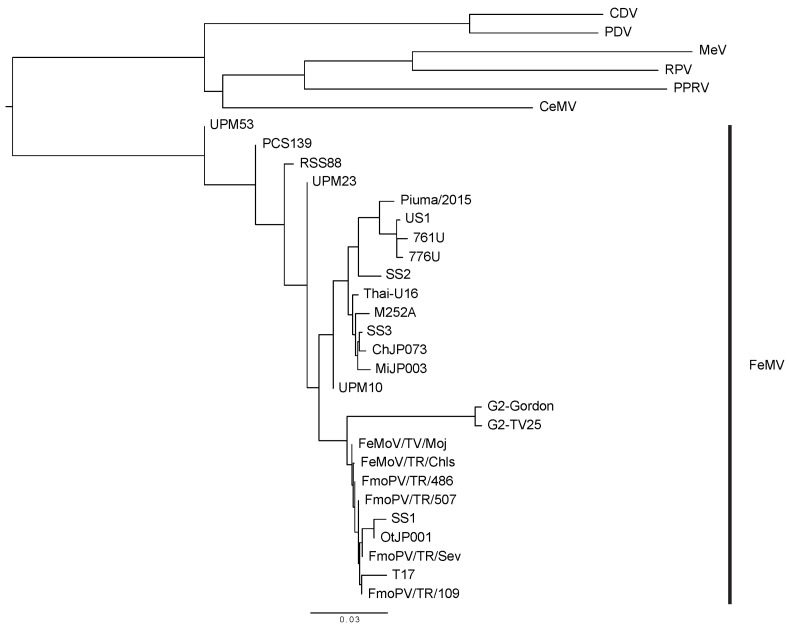
Diagram of the Morbillivirus family. The phylogenetic tree was built after obtaining the RNA polymerase/large protein sequences of the viruses from the NCBI Protein Database. The protein sequences were aligned by using the COBALT multiple alignment tool and the fast-minimum evolution method and visualized using FigTree. The virus names and GenBank accession numbers are: Feline morbillivirus (FeMV) strains TV17 (AVH81382.1), Thai-U16 (AVD98481.1), Piuma/2015 (AMM62640.1), US1 (AMH87247.1), 761U (YP_009512964.1), 776U (AFH55526.1), M252A (AFH55534.1), SS3 (BAR91703.1), SS2 (BAR91698.1), SS1 (BAO58314.1), ChJP073 (BAP74678.1), MiJP003 (BAP74672.1), OtJP001 (BAP74666.1), A1 (AVT56121.1), H10 (AVT56123.1), H1 (AVT56124.1), S1 (AVT56126.1), H3 (AVT56127.1), S2 (VT56128.1), FmoPV/TR/Sev (AMZ80122.1), FmoPV/TR/507 (AMZ80121.1), FmoPV/TR/486 (AMZ80120.1), FmoPV/TR/109 (AMZ80119.1), FeMoV/TR/Moj (ALM58465.1), FeMoV/TR/Chls(ALJ78003.1), PCS139 (AQV13350.1), RSS88 (AQV13353.1), UPM53 (AQV13352.1), UPM10 (AQV13351.1), UPM23 (AQV13349.1), GT2-Gordon (QBC65287.1), GT2-TV25 (QBC65293.1); cetacean morbillivirus (CeMV)—2990 (AYR16899.1), phocine distemper virus (PDV)—Wadden (YP_009177604.1), rinderpest virus (RPV)—LA96 (AEX65767.1), peste-des-petits-ruminants virus (PPRV)—Turkey2000 (CAH61259.1), canine distemper virus (CDV)—PS (AFG24211.1), measles virus (MeV)—Edmonton (AAA75501.1).

Partial L gene sequences of FeMV strains were amplified using RT-PCR from samples such as cat urine, kidney, and blood [2,31]. The large protein sequences from the different FeMV strains were aligned in Figure 2. Whole genome sequences of some viruses were determined by various techniques, such as overlapping RT-PCR amplicons, next-generation sequencing (NGS), and sequence-independent single primer amplification (SISPA) [2,5]. The partial and whole genome sequences known to date are shown in Table 1. MiJP003 is one of the FeMV strains whose complete genome sequence has been determined [2]. Interestingly, the genomic organization and the similarity analysis results showed that the intragenomic region between F and H is different from other strains [2]. This suggests a possible recombination event among the known FeMV strains [2,33].

The rate of FeMV-positive urine samples has varied between studies. One possible explanation is the different clinical and environmental backgrounds of the samples and donors. Stray cats are more easily infected, as they have a higher risk of exposure to infectious agents and conditions, thus the positive rate of the virus in stray cats is higher than that in household cats [10,31,34,35]. Interestingly, unneutered male cats showed a higher risk of FeMV infection than female cats. This may be due to higher activity and aggressive tendencies of male cats, such as territorial fighting and marking behaviors [33,35].

## 3. FeMV Detection Techniques

To isolate new FeMV strains, several techniques have been developed to increase detection efficiency. For instance, a real-time RT-PCR system showed an over ten times higher sensitivity than the conventional RT-PCR method. Using real-time RT-PCR, 25 FeMV positive urine samples were detected out of 166 samples (15.1%). This was about twice the positive rate than the previous study, which showed only six positives out of 82 (7.3%) [31,36]. Furthermore, the reverse transcription loop-mediated isothermal amplification (RT-LAMP) assay has a 100 times higher sensitivity and is time-efficient as compared to conventional RT-PCR [37]. An enzyme-linked immunosorbent assay (ELISA) was also developed and applied for serological detection of FeMV [38]. The purified FeMV P protein was used in the assay as an antigen, because (1) it is important in viral replication, (2) is highly expressed in infected cells, (3) has less conserved gene sequence, and (4) does not need post-translational glycosylation [38]. The P protein-based ELISA assays have been developed for other paramyxoviruses and show higher accuracy and specificity as compared to conventional methods of detection [39,40]. Using ELISA, P protein antibodies were detected in 22 of 100 cats (22%), supporting previous study results [1,32,33].

## 4. Signs of FeMV-Infected Cells and Cats

In vitro, FeMV has been shown to cause cytopathic effects that include cell rounding, detachment, lysis, and syncytia formation in infected Crandall–Reese Feline Kidney (CRFK) cells [1,32,41]. Clinically, FeMV-positive cats have shown urinary tract signs (renal disorders and residue in urine), gastrointestinal signs (anorexia, diarrhea, and vomiting), as well as weight loss, fever, and depression. Additionally, infected cats had decreased red blood cell, hemoglobin, albumin, and urobilinogen counts, as well as higher alanine transaminase, alkaline phosphatase, and bilirubin levels as compared to uninfected cats [10]. However, the authors did not state whether the six FeMV-positive cats were all hospitalized or healthy. Furthermore, they mentioned that some FeMV-positive cats were also positive for other viruses, such as Feline Coronavirus, feline immunodeficiency virus, and feline leukemia virus. Therefore, the signs observed cannot be concluded as caused solely by FeMV.

German strain GT2 (FeMV-GT2), identified in 2019, was isolated from a cat with polyuria–polydipsia syndrome. FeMV-GT2 is phylogenetically distinct and belongs to a different subgroup than other known FeMV strains. FeMV-GT2 can infect cells, such as renal and pulmonary epithelial cells and primary cells from the cerebrum and cerebellum. FeMV-GT2 also infected immune cells, such as CD4^+^ T cells (40–70%), CD20^+^ B cells, and monocytes (20–40%) [46]. However, some of the authors’ observations in this study did not match the previous studies. First, the authors did not observe any cytopathic effects, including syncytia formation, in feline kidney cell lines. Second, the authors suggested that the prevalence of the strain was only 0.83% in urine, which is much less as compared to other studies. This may be due to (1) possible RNA degradation during a few weeks of storage before RNA extraction from the samples and (2) the high genetic diversity between strains [2,33,46,47].

## 5. Virology, Tropism, and FeMV Entry into Host Cells

The in vitro host range of FeMV infectivity has been studied in 32 different cell lines that originated from 13 different animal species, including human, cat, dog, mouse, rat, African green monkey, rabbit, ferret, mink, quail, cattle, horse, and swine [47]. The cells were incubated with the FeMV SS1 strains and cultured for two weeks, and the viral infection was detected by RT-PCR that amplified the L gene. Kidney cell lines derived from both cats and African green monkeys, as well as other feline cell lines, including epithelial, fibroblastic, lymphoid, and glial cells, were susceptible to the viral infection. This suggests the receptor(s) for FeMV, which remain(s) unknown, is(are) ubiquitously expressed, at least in cats. Human cell lines were not susceptible to FeMV, suggesting there is low risk of cross-species transmission between humans and felids [47]. Similarly, transmission between cats remains undetermined. So far, cohabitation has not caused most cats to become FeMV-positive [10]. However, due to the high genetic diversity of the virus and the relatively high mutation rate of the paramyxoviruses, including potential gene recombination, FeMV may have the capacity to adapt to new host species such as humans through physical contact with cats [2,33,47].

Little is known about the specific viral entry mechanism for FeMV. However, host cell receptors such as SLAM (CD150) and nectin-4 are potential candidates, since other morbilliviruses, such as CDV, MeV, RPV, and PPRV use them as their primary receptors for their respective hosts [2,48,49]. For example, MeV suppresses the immune system by binding to the human SLAM on dendritic cells [50,51]. CDV interacts with monkey, dog, and feline SLAM, but less efficiently with the cells expressing human SLAM [52,53,54]. Since receptors are one of the crucial factors to determine the tissue tropism and host range of a virus, it is important to identify the receptor of FeMV [2,53]. Interestingly, the cleavage site of the FeMV F protein is different from the typical cleavage site of other known morbillivirus F proteins. Although immunoblot analysis showed FeMV F cleaved into the typical F_1_ and F_2_ subunits, the FeMV F protein has a single basic proteolytic cleavage site, while other morbillivirus F proteins have multibasic cleavage sites [1,32,55]. This observation suggests that different proteases may cleave the FeMV F protein, which may affect viral entry and host cell tropism.

## 6. Possibility of Persistent FeMV Infection

Several studies have shown evidence of persistent infection with FeMV [13,32,33,43,46]. For example, FeMV strain US1 was obtained from a male domestic cat in 2013, and the identical strain was detected in the same cat 15 months later based on amplification and sequencing of the H gene [43]. Furthermore, almost half of the infected cats (14 out of 29) were positive not only for RNA, but also for antibodies against the N protein [33]. Further, two cats infected by FeMV strain GT2 shed the virus in their urine for up to several years [46]. These results suggest that persistent FeMV infection is possible. Interestingly, while cat urine (50.8%) and kidney (80.0%) samples were found FeMV-positive as determined via nested RT-PCR targeting the L gene, blood samples were all FeMV-negative [35]. This suggests that the cats were not viremic when the samples were collected. These observations suggest that FeMV either has a long incubation period or a short viremic duration [35]. Another possibility is that during early stages of infection, FeMV in circulation may infect lymphocytes and is below the threshold of PCR detection [56,57]. Overall, the pathogenesis of FeMV remains not well understood. Further studies will be required with a larger sample size and various incubation periods to understand persistent FeMV infections.

## 7. Controversies of FeMV Studies

A controversy surrounding FeMV research is whether the virus is involved in tubulointerstitial nephritis (TIN). This is one of the primary causes of renal failure, which can lead to and may trigger chronic kidney disease (CKD). This is one of the most common metabolic diseases of cats, particularly for older cats, frequently causing feline death [42,58,59]. There has been a suggested association between FeMV and TIN after the discovery that seven out of 12 FeMV-infected cats had TIN [1]. Additionally, four of the fixed kidney tissues from ten cats with nephritis (40%) were FeMV-positive [31,60]. Furthermore, a significant association between FeMV infection and TIN was found based on immunohistochemistry (IHC) [60]. The pathology observed in 38 kidney tissue samples was consistent with chronic kidney disease, including interstitial cell infiltration, glomerulosclerosis, tubular atrophy, and fibrosis. The authors also compared FeMV-positive and negative samples, scored and statistically evaluated the correlation between FeMV infection and TIN, and found particular statistical significance in tubular atrophy, luminal expansion, urinary casts for renal tubules, inflammatory cell infiltration, and fibrosis in the interstitial areas. The differences were significant for thickness of capillaries and glomerulosclerosis in renal tubules [60]. On the other hand, other conducted studies were unable to find a clear statistical relationship between cat nephritis and FeMV infection [10,12,32,34,35,44]. FeMV, however, might be involved in CKD or lower urinary tract diseases (LUTD), based on the reported IHC detected in 19 cat kidney tissues of FeMV-infected cats [33]. However, the authors proposed that FeMV may not necessarily cause feline urinary tract diseases, but simply act as a helper or bystander [33]. Several possible explanations of this controversy have been proposed. First, some feline chronic diseases, including TIN and CKD, can still develop when no FeMV RNA is detected in urine. Second, the research that showed no clear association between FeMV and TIN or CKD might have chosen indirect markers for detection. Finally, the primers used for FeMV detection may be relatively poorly optimized [60]. Another controversy in the field is the potential cross-reactivity between FeMV and CDV. Sakaguchi et al. showed immunoreactivity of the anti-FeMV N antibody to CDV N, and of anti-CDV dog serum to FeMV [32]. However, other studies did not find any cross-reactivity between these two viruses using an immunofluorescence (IF) test for anti-FeMV serum binding to CDV N, an ELISA assay to test cross-reactivity between CDV P and FeMV P, and an RT-LAMP assay using the RNA extracted from CDV-infected Vero cells [33,37,38].

## 8. Conclusions

Cats are among the most common household pets. Feline kidney diseases such as TIN and CKD are among the leading causes of death in domesticated cats, particularly in geriatric cats. Although the link between FeMV and kidney diseases has not been clearly defined, an association is possible, even if it is not causal. It is possible that some cats establish persistent FeMV infection, shedding FeMV RNA in their urine for extended periods of time. Moreover, considering the high genetic diversity of FeMV, there is a possibility for cross-species infections. Therefore, FeMV research may have significance beyond feline health. Since FeMV is a relatively newly identified virus, currently, there are not enough case studies or clinical data available. Therefore, further studies with larger sample numbers or full genome sequences of the identified strains would be beneficial to understand the effects of FeMV in the worldwide feline health. 

## Figures and Tables

**Table 1:** Reported FeMV complete/partial sequences.

Virus	Strain	Country	Reference	Sequence	GenBank Accession No.
FeMV	761U	Hong Kong	[1]	Complete	JQ411014
	776U				JQ411015
	M252A				JQ411016
	SE4	Japan	[31]	Partial (L gene)	AB828138
	CL5				AB828139
	SE7				AB828140
	SE14				AB828141
	MS25				AB828142
	MS26				AB828143
	SS1	Japan	[32]	Complete	AB910309
	SS2				AB910310
	SS3				AB910311
	OtJP001	Japan	[2]	Complete	AB924120
	MiJP003				AB924121
	ChJP073				AB924122
	Piuma/2015	Italy	[42]	Partial (L gene)	KT306750
			[5]	Complete	KT825132
	US1	US	[43]	Complete	KR014147
	BR1	Brazil	[12]	Partial (L gene)	KX452077
	FmoPV/TR/109	Turkey	[10]	Partial (L gene)	KU053510
	FmoPV/TR/486				KU053511
	FmoPV/TR/507				KU053512
	FmoPV/TR/Sev				KU053513
	A1	UK	[44]	Partial (L gene)	MG640027
	S9				MG640028
	H10				MG640029
	H1				MG640030
	S6				MG640031
	S1				MG640032
	H3				MG640033
	S2				MG640034
	TV17	Germany	[45]	Complete	MG563820
	1073U	Italy	[34]	Partial (L gene)	N/A
	434K				
	1568K				
	Tremedino/2018	Italy	[41]	Complete	MK088516
	Pepito/2018				MK088517
	UPM23	Malaysia	[35]	Partial (L gene)	KU646847
	PCS139				KU646848
	UPM10				KU646849
	UPM53				KU646850
	RSS88				KU646851
	UPM23			Partial (N gene)	KU646852
	PCS139				KU646853
	UPM10				KU646854
	UPM53				KU646855
	RSS88				KU646856
FeMV-GT2	Gordon	Germany	[46]	Complete	MK182089
	TV25				MK182090

* N/A indicates not available.
